# Identification of Protein Tyrosine Phosphatase Receptor Gamma Extracellular Domain (sPTPRG) as a Natural Soluble Protein in Plasma

**DOI:** 10.1371/journal.pone.0119110

**Published:** 2015-03-16

**Authors:** Elisabetta Moratti, Marzia Vezzalini, Luisa Tomasello, Davide Giavarina, Claudio Sorio

**Affiliations:** 1 Department of Pathology and Diagnostics, University of Verona, Verona, Italy; 2 Clinical Chemistry and Hematology Laboratory St. Bortolo Hospital, Vicenza, Italy; Thomas Jefferson University, UNITED STATES

## Abstract

**Background:**

PTPRG is a widely expressed protein tyrosine phosphatase present in various isoforms. Peptides from its extracellular domain have been detected in plasma by proteomic techniques. We aim at characterizing the plasmatic PTPRG (sPTPRG) form and to identify its source.

**Methodology/Principal Findings:**

The expression of sPTPRG was evaluated in human plasma and murine plasma and tissues by immunoprecipitation and Western blotting. The polypeptides identified have an apparent Mr of about 120 kDa (major band) and 90 kDa (minor band) respectively. Full length PTPRG was identified in the 100.000×g pelleted plasma fraction, suggesting that it was present associated to cell-derived vesicles (exosomes). The release of sPTPRG by HepG2 human hepatocellular carcinoma cell line was induced by ethanol and sensitive to metalloproteinase and not to Furin inhibitors. Finally, increased levels of the plasmatic ∼120 kDa isoform were associated with the occurrence of liver damage.

**Conclusions:**

These results demonstrate that sPTPRG represent a novel candidate protein biomarker in plasma whose increased expression is associated to hepatocyte damage. This observation could open a new avenue of investigation in this challenging field.

## Introduction

Protein tyrosine phosphatases gamma (PTPRG) belongs to a family of enzymes that remove phosphate groups from phosphotyrosine residues of specific intracellular targets. PTPRG maps to chr 3p14.2/21, and its mRNA (5787 bp coding sequence) encodes for a transmembrane protein with three different domains in the extracellular amino terminus region: a carbonic anhydrase-homologous, a fibronectin type III domain, a transmembrane region and an intra-cytoplasmatic region composed of two intracellular PTPase catalytic domains. As broadly expressed enzyme, PTPRG behaves as a tumor suppressor gene subjected to loss of heterozygosity deletion, hypermethylation, and point mutation [1–3]. In spite of the availability KO mice models [4,5] and of several studies carried out to investigate its expression and role in healthy and diseased tissues[6,7], PTPRG is an orphan receptor which signaling pathways are still largely unknown although the polycystin-1 C-terminus, ABL and BCR/ABL have been reported to behave as PTPRG substrates [8,9]. It is known that PTPRs ectodomains can be cleaved off by specific protease and remain as non-covalently bound subunits extracellularly [10]. In addition these ectodomains may be shed, and thus may have a function by themselves acting as ligand, independently of the catalytic activity of the enzyme and may modulate intracellular PTP activity. Indeed, PTPRF (LAR) is cleaved intracellularly into two subunits[11]. Proteolytic processing of other members of the PTPR family has been reported [12–15]. Among these, it is known that three isoforms of PTPRZ, the only other member of class V PTPRs beside PTPRG, are generated by alternative splicing from a single *Ptprz* gene in the rat [16]. Ptprz-A and Ptprz-B can undergo further processing by metalloproteinase-mediated ectodomains shedding, which releases the extracellular fragment Z_A/B_-ECF (extracellular fragment), from the cell surface and produces the membrane-tethered counterpart ZΔE. ZΔE is digested by PS/γ-secretase, and the cytoplasmic fragment Z-ICF (intracellular fragment). Z-ICF can be released from the plasma membrane and is detected not only in the cytoplasm but also in the nucleus, suggesting a novel signaling pathway driven by PTPRZ [17]. Finally, four different isoforms of PTPRG, one of which is a soluble form, have been identified in rodents. PTPRG isoforms are derived from alternative splicing, and are designated as Ptprg-A (full length isoform), Ptprg-B (lacking the intracellular juxtamembrane 29 amino acid) [18], Ptprg-C that have only one phosphatase domain and Ptprg-S an extracellular variant of the protein that is secreted into culture medium when expressed in COS7 cells[19]. The presence of different PTPRG protein isofoms was suggested in human and mice tissues on the basis of their different reactivity with specific antibodies [6,7] as well as on the identification of PTPRG–derived peptides circulating in the plasma [20–22]. Here we describe for the first time a biochemical characterization of circulating PTPRG isoforms and provide evidence that hepatocytes represent a major source of sPTPRG. We also found a positive correlation of sPTPRG with high plasmatic levels of biomarkers associated to liver damage.

## Materials and Methods

### Antibodies

**Fig 1:**
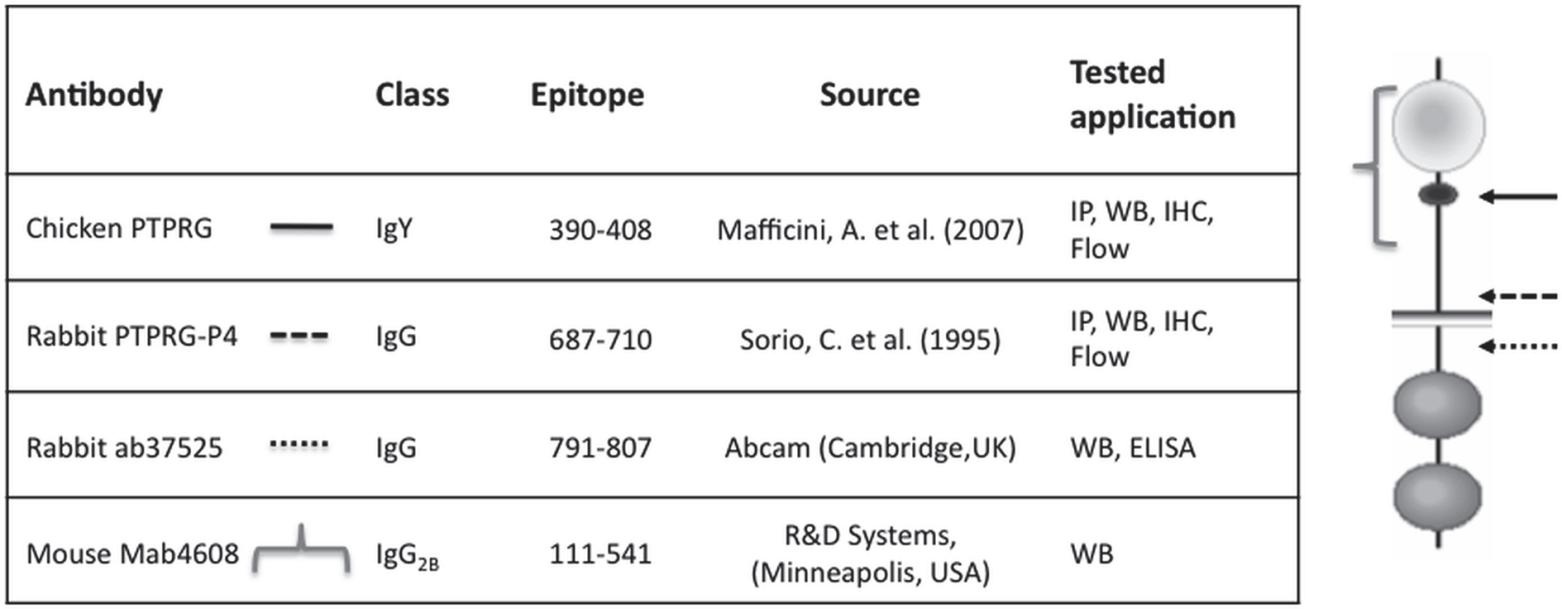
Schematic representation of antibodies used. Epitope: aminoacid number of epitope (NCBI protein accession number P23470), Tested application: IP = immunoprecipitation, WB = Western blot, IHC = immunohystochemistry, Flow = flow cytometry. Right image: approximate position of the epitopes recognized by selected antibody within the predicted protein sequence.

**Fig. 1** summarizes the features of the antibodies used in this work.

### Human and murine plasma

Plasma was obtained from leftover specimens, remnants of specimens collected for routine clinical care into lithium heparin vacuum tubes, centrifuged at 2600×g for 10 minutes and frozen at -80°C within 3 hours until use. Alanine aminotransferase (ALT) was measured by a chemistry analyzer ADVIA 2400 (Siemens Healthcare Diagnostics, Terrytown, USA). The specimens from Clinical Chemistry and Hematology Laboratory St. Bortolo Hospital were made anonymous before freezing and provided to the investigators without identifiers. The local Ethic Committee for Clinical Trials (CESC) of Vicenza Area (Protocol 8/2014) approved the study.

### Mice perfusion and tissue collection

Male C57BL/6J mice, 5–8 weeks of age (The Jackson Laboratory, Bar Harbor, ME, USA) were maintained under pathogen-free conditions. This study was carried out in strict accordance with the recommendations in the Guide for the Care and Use of Laboratory Animals of the National Institutes of Health. The Committee on the Ethics of Animal Experiments of the University of Verona (CIRSAL- Centro Interdipartimentale di Servizio alla Ricerca Sperimentale 11105, Permit Number 187/2012-B) approved the protocol. All efforts were made to minimize suffering: the mice were anesthetized with an i.p. injection of Ketamine/Xilazina (80–100mg/kg Ketamin and 10mg/kg Xilazin in PBS). Following abdominal incisions, the heart of the mice were perfused with Phosphate Buffer Saline (PBS), 22 mM glucose, 1 mM CaCl_2_ and 1 mM MgCl_2_ through the left ventricle of the heart using Masterflex Pump Controller (Cole Parmer Instrument Co., Chicago, IL, USA) at a flow rate of 4 mL per minute. This flow rate approximates the murine physiological blood pressure. When all the blood was replaced with the saline solution organs (brain, thymus, heart, lung, kidney, spleen, liver and pancreas) were removed, snap-frozen with dry ice, and stored at -80°C.

### Purification of soluble PTPRG and production of recombinant sPTPRG

A recombinant sPTPRG derivative consisting of the extracellular domain without transmembrane segment and cytoplasmic tail, was expressed in HEK 293F cells. To obtain the PTPx-FcIgG3 construct, the cDNA fragment corresponding to extracellular domains of PTPRG 1 through 736 was cloned into pCEP4 plasmid carrying a mouse IgG3 domain between KpnI and BamHI and was inserted in frame between Bcl I (inside IgG3 domain) and Kpl I. Transfected HEK 293F cells were cultured in RPMI 1640 containing 10% heat-inactivated Fetal Calf Serum (FCS), 4mM glutamine, and 0,5 mg/mL of hygromycin (Invitrogen Milan, Italy) as selective agent or cultured in serum free medium CD293 (Gibco, Milan, Italy) with 50 μM β-mercaptoethanol and 0,5 mg/mL of hygromycin (Invitrogen, Milan, Italy). All the cultured cells were grown at 37°C, in 5% CO_2_. The recombinant ECD-PTPRG-Fc fusion protein was purified from serum free medium cultured cell by affinity chromatography using protein-G Sepharose according to the manufactory protocol of HiTrap Protein G HP, 1 ml (GE Healthcare, Milan, Italy). The yield was around 0,2 mg/ml for >80% of purity.

### Processing of cells and tissues

Human hepatocellular carcinoma cell line (HepG2, ATCC HB 8065) was cultured in EMEM containing 2mM glutamine, 1mM Na pyruvate and 10% FCS or, when appropriate, in FCS free medium in the presence of 50 μM Furin inhibitor II (Calbiochem, Merck KGaA, Darmstadt, Germany) or alternatively 12.5 μM GM6001 (Ilomastat) (Millipore, 290 Concord Road, Billerica, MA 01821 USA). About 2x10^7^ cells/mL were solubilized in lysis buffer (LB) composed of 50 mM Tris (pH 7.4), 1% Triton X-100, 150 mM NaCl, 200 μM NaVO_4_, 1 mM EDTA, 1 mM DTT, 1 mM NaF, Complete EDTA-free Protease Inhibitor Cocktail Tablets (Roche, Germany). Cells were incubated for 20 min. in a rotating wheel at 4°C and then centrifuged for 30 min at 4°C at 13000×g in a Biofuge Pico, (DJB Labcare Ltd. UK). A number of 10–20 serial slices (30 μm-thick) from frozen murine tissues were lysed in LB. Quality of the tissue was checked with Hematoxyilin-Eosine staining. Equal protein amounts were mixed with sample buffer (SB), subjected to SDS-PAGE and electroblotted on nitrocellulose membrane.

### Exosomes Purification from Blood

10 mL of fresh blood from two healthy donors were collected in EDTA coated tubes. The tubes were inverted to mix the samples with EDTA and placed upright at room temperature for 10–60 minutes. Then blood was centrifuged at 2,000×g for 15 minutes at 4°C and the top layer containing plasma was collected in 15 mL tubes and kept on ice. The plasma was diluted with an equal volume of 1x PBS and centrifuged at 2,000xg for 30 minutes at 4°C. For each donor, plasma was transferred into an ultracentrifuge tube (and 1x PBS was added to a total volume of 15 mL) and centrifuged at 12,000×g for 45 minutes at 4°C. The supernatant was transferred to a new ultracentrifuge tube and centrifuged 2 hours at 110000×g at 4°C. The pellet formed by all secreted extracellular vesicles was resuspended in 15 mL of 1xPBS and centrifuged at 110,000×g for 1 hour at 4°C for two times. Finally, the pellet was lysed in an appropriate volume of RIPA buffer [50 mM Tris-Cl pH 7.5, 150 mM NaCl, 1% Nonidet P-40, 0,5% Sodium Deoxycholate, 0,1% SDS, 200 μM Sodium Orthovanadate, 1mM DTT, 10 mM NaF, 1mM EDTA, and 1X Complete EDTA-free Protease Inhibitor Cocktail Tablets (Roche)], mixed to 0.25 volumes of sample buffer (160 mM Tris, 20% Glycerol, 5% β-mercaptoethanol, 4% SDS, 0.01% bromophenol blue) and subjected to Western Blotting (WB).

### Protein precipitation from HepG2 supernatants

Cells were seeded in a 75 cm^2^ flask and cultured in EMEM (BioWhittaker, Lonza), 2mM Ultraglutamine and 10% FBS (Lonza Group Basilea, Switzerland) until the confluence was 70–80%. Then cells were cultured in 10 mL serum free medium in presence or not of 50 mM EtOH (Sigma-Aldrich Milan, Italy) and collected the supernatants after overnight incubation. We added 1 volume of 100% (w/v) TCA to 4 volumes of cell supernatants and incubated on ice for 2 hours in stirring. After a centrifugation at 13000×g for 15 min, we collected the whitish, fluffy pellets and washed twice with cold acetone. The dried pellets were resuspended in an appropriate volume of sample buffer (160 mM Tris, 20% Glycerol, 5% β-mercaptoethanol, 4% SDS, 0.01% bromophenol blue) and subjected to WB.

### Immunoprecipitation from plasma

Plasma samples from blood donors and patients were diluted 1:5, 1:10, 1:20 and 1:40 in PBS/TX100 1% to a final volume of 500 μl, respectively. A 5 μl volume of CNBR Sepharose 4B conjugated with Rb anti-P4 and its control (RbIgG) were added to samples stirred for three hours in a rotating wheel. Then, beads were washed three times with PBS and the pellet was mixed with SB and denatured at 95°C for 10 minutes.

### Immunoprecipitation from cell lysates

Total protein content was assessed by Bradford assay (Sigma, Milan, Italy). Serum-free conditioned medium (SFCM) or cell lysates (250 μg of total protein in each sample) were added with 3 μg of specific antibodies and incubated for 3h at 4°C. A 20 μl volume of protein G-Sepharose beads (Sigma St. Louis, MO, USA) previously washed once in Tris-buffered saline (TBS) and three times in LB or serum free medium was added to the samples incubated for 1 h at 4°C under gentle rocking. Then the sample was washed three times in TBS buffer and the final pellet was resuspended in SB and subjected to analysis. All the experiments shown were performed a minimum of 2 times with consistent results.

### siRNA transfection

Small interfering RNA (siRNA) targeting PTPRG (siPTPRG, n° s-11550) and negative control (scramble) siRNAs were purchased from Applied Biosystems Life Technologies (Foster City, California, United States). To silence PTPRG expression in vitro, 1x10^5^ cells /mL were transfected with 30 nM of siRNAs using siPORT NeoFX Transfection Agent (Applied Biosystems Life Technologies, Foster City, California, United States), according to the manufacturer's instructions. Cells were cultured for approximately 60 hours in RPMI 1640, 2mM Ultraglutamine and 10% FBS (Lonza Group Basilea, Switzerland) and in serum depleted medium for 12 hours, washed twice with cold TBS and lysed. Protein extraction of serum free supernatants of treated cells was carried out by precipitation with 20%TCA and pellets were washed twice with 100% cold acetone. Dried pellets were directly resuspended in sample buffer (160 mM Tris, 20% Glycerol, 5% β-mercaptoethanol, 4% SDS, 0.01% bromophenol blue) and 30 μg of quantified proteins by Bradford assay were subjected to WB. All the experiments shown were performed a minimum of 2 times with consistent results.

### Deglycosylation

Deglycosylation of purified recombinant extracellular domain of PTPRG linked to Fc (ECD-PTPRG-Fc) and the immunoprecipitated plasmatic PTPRG was carried out with the use of PNGase F (Boehringer, Mannheim, Germany) according to the manufacturer’s protocol.

### Biotin labeling of cell-surface proteins

After three washes with 10 mL 1xPBS pH 8.0, adherent HepG2 cell line treated or not with 50 mM Ethanol (EtOH) were incubated with 10 mM non-cell permeable thiol-cleavable sulfo-N-hydroxysuccinimide-SS-biotin (EZ-Link sulfo-NHS-SS-Biotin, Thermo Scientific) for 30 min in stirring at 4°C. To quench the biotinylation reaction, cells were flushed three times with glycine 100 mM in PBS pH 8.0. Cells were lysed in RIPA buffer, protein samples were immunoprecipitated with anti-P4 antibody and then subjected to WB analysis. All the experiments shown were performed a minimum of 2 times with consistent results.

### Western blot

Cell lysates, serum free supernatants and tissue lysates were separated on 6–7,5–10% SDS-PAGE in a Mini Protean 3 Apparatus (Biorad, Milan, Italy) and then electroblotted onto nitrocellulose membrane (Millipore Corp., Bedford, MA) previously blocked with 3–5%BSA in TBST (0.05% Tween-20) and probed with suitable primary polyclonal or monoclonal antibodies at 1 μg/mL in 1% BSA TBST buffer overnight. After washes in TBST, the membrane was then incubated with the following secondary antibody: 1) donkey anti-rabbit IgG-HRP (GE Healthcare, Little Chalfont, UK), 2) goat anti-mouse-HRP conjugated (GE Healthcare, Little Chalfont, UK) or 3) with anti mouse Fc-HRP conjugated (NOVUS biological, Littleton, CO, USA) for 1 h, respectively. After membrane washing, the signal was detected with the enhanced chemiluminescence kit (Millipore Corp., Bedford, MA). To perform the quantitative analysis of signal intensity the Quantity One software 4.6.5 (BioRad, Munich, Germany) was used. The average density is expressed in arbitrary units (Adj volume INT*mm^2^). All the experiments shown were performed a minimum of 2 times with consistent results.

## Results

### Detection of PTPRG isoform in plasma/serum

**Fig 2:**
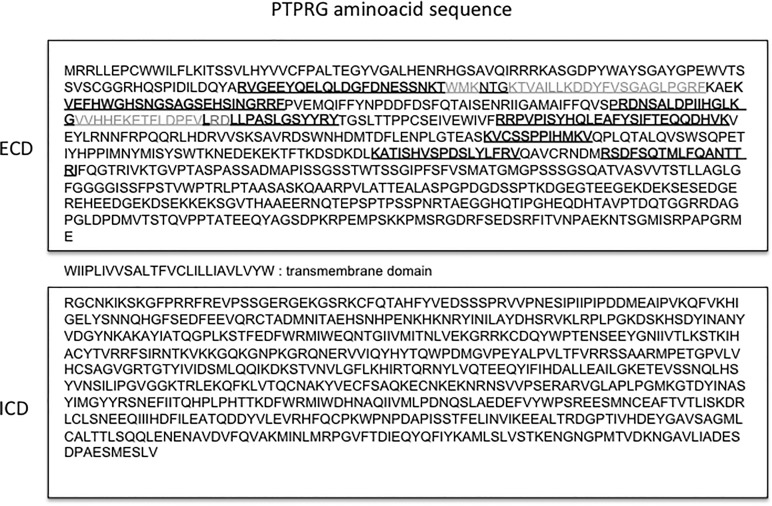
Localization of PTPRG-derived peptides. Schematic representation of peptides identified by large-scale proteome analysis and reported in the literature[20–22]. In grey are the sequences found in all the studies. ECD: extracellular domain, ICD: intracellular domain.

Peptides derived from PTPRG extracellular domain was described in human plasma[20–22]. **Fig. 2** summarizes these data and highlights the position of the reported peptides in relation to the predicted PTPRG aminoacid sequence (NCBI protein accession number P23470). All the peptides identified in the plasma fraction belong to the extracellular domain. In order to confirm the presence and characterize the features of PTPRG in plasma (sPTPRG) we performed immunoprecipitation (IP) of sPTPRG followed by Immunoblotting detection with the same antibody capable of recognizing both human and murine PTPRG.

### PTPRG isoforms

**Fig 3:**
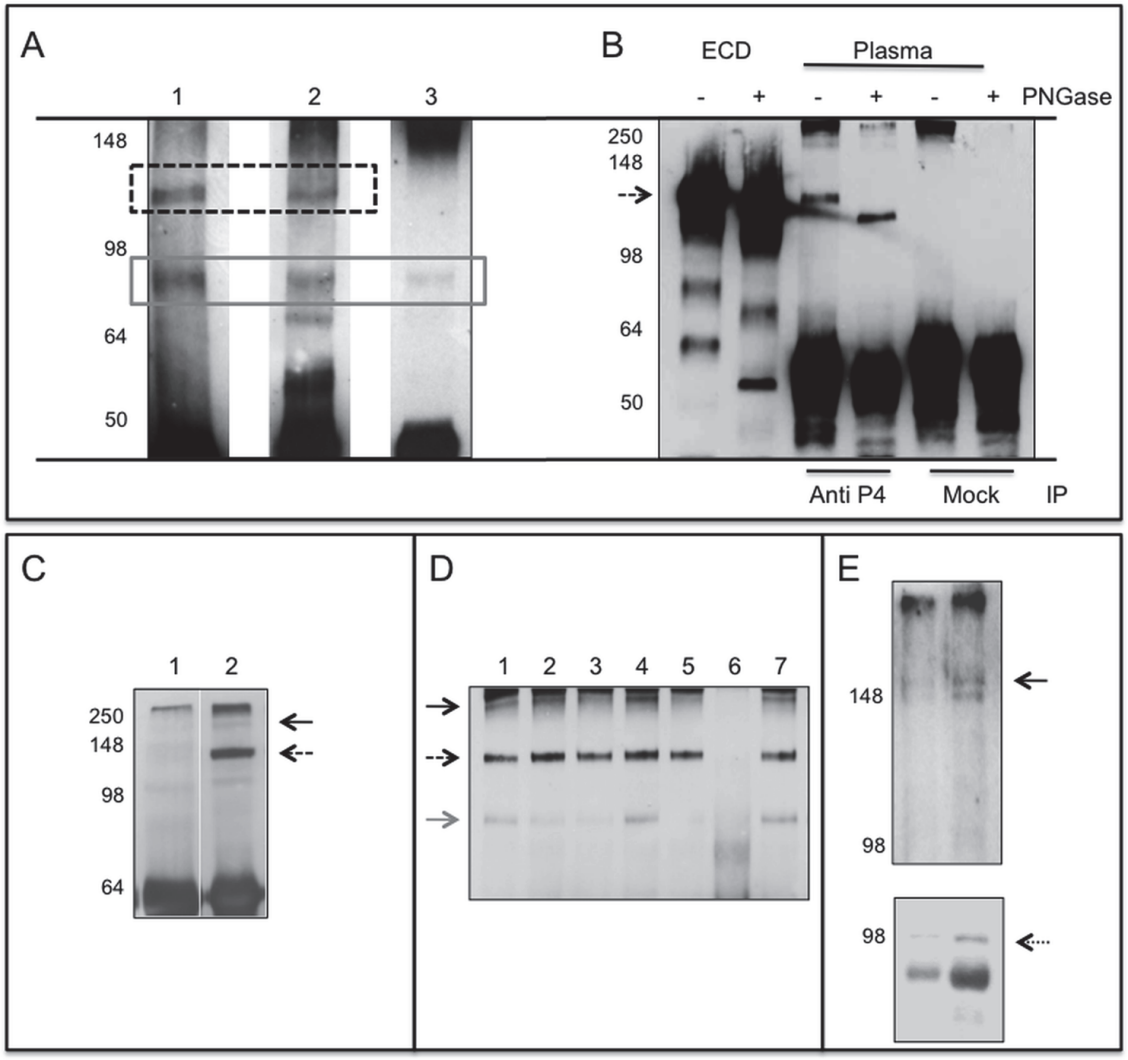
PTPRG plasmatic isoforms. **Panel A**: Immunoblotting of human plasma samples immunoprecipitated (IP) with Rb anti-P4 and blotted against: Lane 1: Rb anti-P4, lane 2: mouse monoclonal R&D, lane 3: rabbit polyclonal Abcam. Dashed box: ∼120 kDa PTPRG isoform, gray solid box: ∼90 kDa isoform. **Panel B**: Immunoblotting with Rb anti-P4 of purified recombinant PTPRG-ECD-Fc lanes 1,2 and immunoprecipitated plasma samples with anti-P4 and RbIgG isotype control (Mock). Sample treated with the deglycosylating enzyme PNGase F are labeled with + sign. Dashed arrow: PTPRG ∼120 kDa isoform. Immunoblotting with Rb anti-P4 of anti-P4 IP human plasma (**Panel C**) and mouse serum (**Panel D**) of samples diluted 1:20 in PBS/Tx100 1%. **Panel C**: Lane 1: Immunoprecipitation with isotype control; Lane, 2: Rb anti-P4. **Panel D**: Lane 1–5 and 7: IP with Rb anti-P4. Lane 6: IP with RbIgG isotype control. Black arrow: full-length protein, dashed arrow: ∼120 kDa isoform, gray arrow: ∼90 kDa isoform. Left: molecular weight markers. **Panel E**: Plasma exosomes purified from two individuals were blotted with Rb-anti-P4 (upper panel) and anti ALIX rabbit antibody (lower panel, dotted arrow), an exosome marker. A band corresponding to full-length PTPRG is detectable in both samples (black arrow). Left: molecular weight marker.

The ∼120 kDa protein is recognized by antibodies generated against the extracellular domain (**Fig. 2 and Fig. 3A**, lanes 1,2) but not by the antibody directed to an intracellular epitope (**Fig. 3A**, lane 3) suggesting that the transmembrane domain was retained in the 90 kDa isoform. The R&D antibody (spanning residues 111–541) recognized also a band around 80 kDa of unknown origin and specificity.

### PTPRG glycosylation

Based on sequence analysis we predicted an extracellular domain form of 81 kDa while the protein we detected has an apparent Mr of about 120 kDa. This discrepancy could be explained by the occurrence of glycosylation, a typical feature in extracellular domains of many receptor-like polypeptides and has been described to occur in baculovirus-expressed PTPRG[18]. Glycopeptidase F (PNGase F) is a deglycosylating enzyme that cleaves asparagines linked to high mannose as well as hybrid and complex oligosaccharides from glycoproteins and deaminates the asparagine to aspartic acid, but leaves the oligosaccharide intact. The treatment caused a reduction of Mr from ∼120 to ∼100 kDa. The same shift in Mr was observed using the 293 cell line-expressed PTPRG-ECD-Fc (**Fig. 3B**). The slightly higher Mr of the recombinant protein is due to the presence of a fusion cDNA with a murine Fc tag.

**Fig. 3C** displays a representative result obtained analyzing 24 human plasma samples where PTPRG was immunoprecipitated with anti-P4 antibody and subjected to WB using the same anti-P4 antibody and two more antibodies raised against extra and intracellular domains while **Fig. 3B** shows the result derived from the analysis of serum samples from six mice. A major band with an estimated Mr of about 120 kDa was consistently detectable. Another ∼90 kDa band was detectable in some preparation (**Fig. 3D**). Since we noticed also the presence of a faint band corresponding to the putative full-length (FL) PTPRG we investigated if this could derive from exosomes which are 30–100 nm size cell-derived vesicles present in biological fluids [23]. We analyzed the presence of this band in the pellets from plasma samples subjected to ultra-centrifugation at 100000×g. The band corresponding to the full length PTPRG protein could be detected in the pelleted fraction expressing also the exosome-associated marker ALIX (**Fig. 3E)**. Both ∼120 kDa and the smaller, less consistently detectable ∼90 kDa isoforms, are still present in the post-ultracentrifugation plasmatic fraction (not shown). [20–22]

### Expression in murine tissues

**Fig 4:**
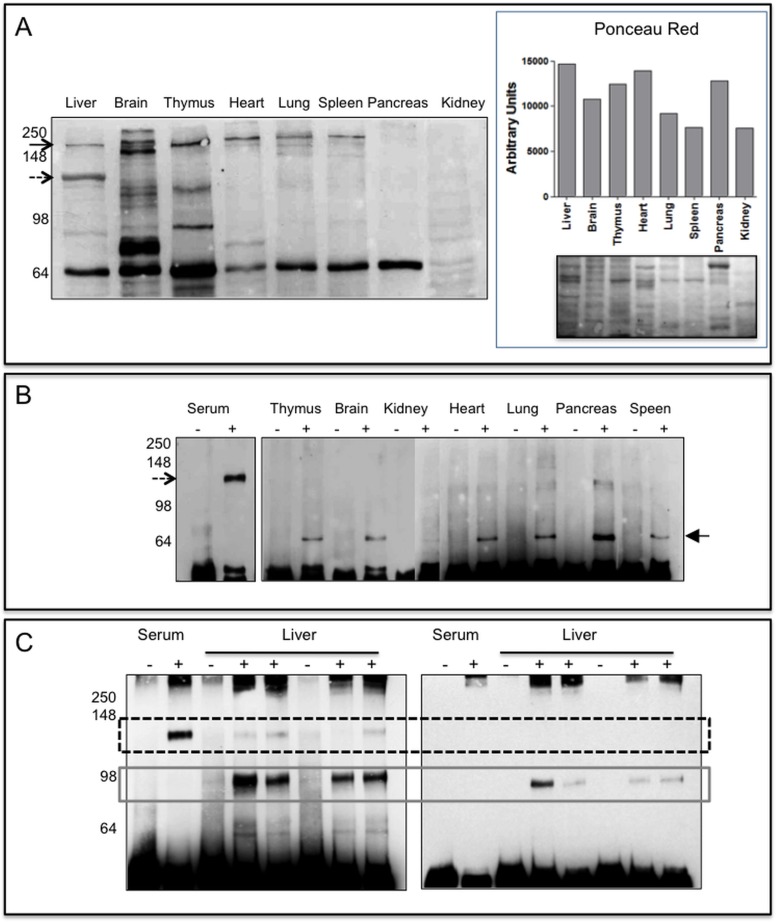
PTPRG expression in murine tissues. **Panel A (left):** 20μg of lysates from perfused tissues were loaded on 10% SDS-PAGE, separated by gel electrophoresis and then blotted onto a PVDF membrane successively incubated with Rb anti-P4 antibody. Black arrow: full-length protein, dashed arrow highlights the 120 kDa protein detectable in liver lysates. On the right it is reported the densitometric analysis of the same samples stained with Ponceau Red and the corresponding image. **Panel B**: Immunoblotting reacted with Rb anti-P4 antibody of immunoprecipitated (IP) murine serum and tissue lysates (IP from 250 μg of tissue lysates). Lanes-: IP with Protein G Sepharose rabbit IgG isotype control. Lanes +: IP Protein G Sepharose Rb anti-P4. Dashed arrow: ∼120 kDa isoform, black filled arrow: ∼70 kDa isoform, maybe an intracellular, immature form of a soluble protein, or of a fragment of the full-length protein also present in Panel A. Lack of detectable amounts of full length protein is likely associated to residual proteolysis occurring during the IP step. **Panel C:** Immunoprecipitation from mouse serum and liver lysates performed with anti P4 antibody (+) or with RbIgG isotype (-). Left: WB performed with Rb anti-P4 reacting against an epitope located within the extracellular domain of human and murine PTPRG. Right: WB with polyclonal anti PTPRG (791–807 Abcam) reacting against an epitope located in the intracellular domain. Dashed box: ∼120 kDa and grey solid box: ∼90 kDa isoforms, respectively.

In order to identify the tissue source of sPTPRG we removed any trace of plasma performing organ perfusion in mice in order to subtract the contribution of the plasma present in vascolarized tissues. The full-length protein is detectable in almost all the tissues examined except pancreas and kidney while an immunoreactive ∼120 kDa protein was readily detectable only in the liver. Among other bands variably expressed in tissues a ∼70 kDa one was consistently detected in all tissues except kidney (**Fig. 4A and B**)

In order to identify additional sources of the 120 kDa antigen, we immunoprecipitated PTPRG from a larger amount of cell lysate (250 μg) and performed WB on this material (**Fig. 4A and B**). Only lung, pancreas and liver express barely detectable levels of the ∼120 kDa isoform confirming the liver as a main source of sPTPRG. Moreover, immunoblot assay confirm the presence of an approximately 70 kDa band in all the tissues investigated (with the exception of kidney and liver) that needs to be better characterized.

We then focused on liver and performed immunoprecipitation with anti-P4 antibody followed by WB with the same antibody and Abcam antibody recognizing the juxtamembrane intracellular domain. Two major bands of approximately ∼120 and ∼90 kDa were identified (**Fig. 4C**, left panel). The ∼90 kDa band reacted with an Abcam antibody recognizing the juxtamembrane intracellular domain, thus indicating that epitopes located on both sides of the transmembrane region are present in this isoform that is absent in the ∼120 kDa isoform (**Fig. 4C**, right panel).

### HepG2 release sPTPRG

**Fig 5:**
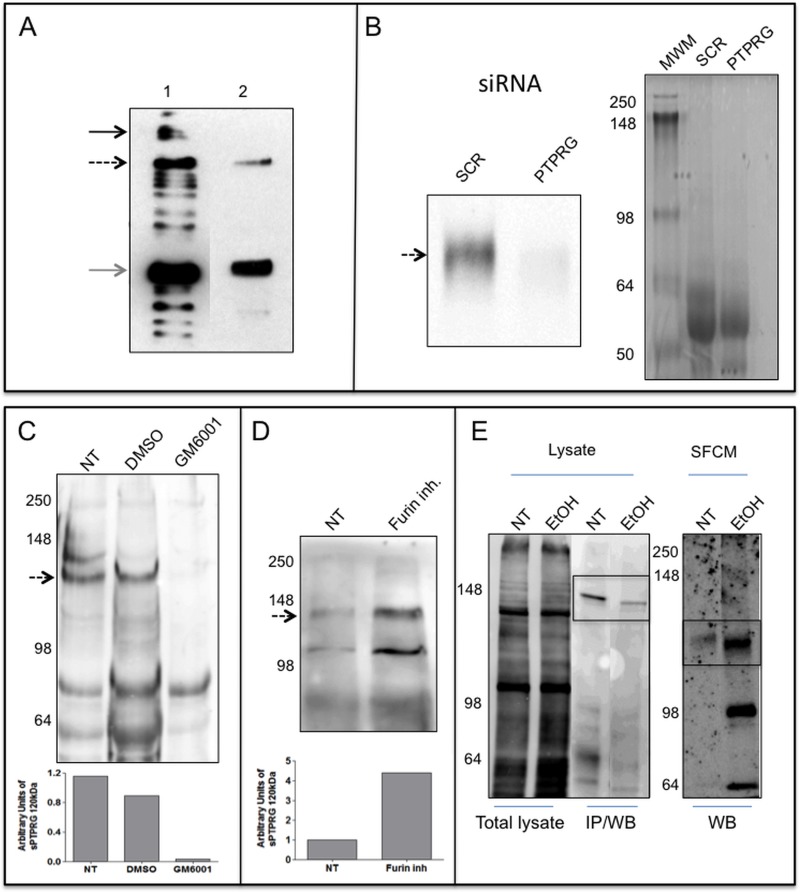
Expression and release of PTPRG by HepG2 cells. **Panel A**: WB with Rb anti-P4 of total lysate and supernatant of HepG2 cell line. Lane 1: 10 μg total cell lysate of HepG2 cell line. Lane 2: serum-free conditioned medium of HepG2 after 100000×g ultra-centrifugation and TCA precipitation. Black arrow: full-length protein, dashed arrow: ∼120 kDa isoform, gray arrow: ∼90 kDa isoform. **Panel B**: WB with Rb anti-P4 of serum-free conditioned medium of HepG2 cells showing down regulation of sPTPRG by siRNA (siRNA PTPRG) in comparison with a scrambled sequence (SCR). Coomassie blue staining of serum-free conditioned samples demonstrating the loading on comparable amounts of material in both lanes. **Panel C**: serum-free conditioned medium (SFCM) of HepG2 cells. Left: Molecular weight marker. NT: untreated cells, DMSO: cells treated with vehicle (DMSO), or overnight with 12 μM metalloproteinase inhibitor GM6001 (Ilomastat). Densitometric analysis of sPTPRG 120 kDa isoform related to a common aspecific band approximately at 70 kDa present in all samples (**below**). **Panel D**: cells treated overnight with 50 μM furin inhibitor. Dashed arrow: ∼120 kDa isoform. Below is the densitometric analysis expressed as fold increase versus control of the ∼120 kDa band normalized against the total protein load. WB was performed with a chicken anti-PTPRG antibody[27]. **Panel E**: WB with streptavidin-HRP of cell-surface biotinylated HepG2 cells untreated (NT) or treated (EtOH) for 16 hours with 50 mM ethanol, lysed and immunoprecipitated with anti-P4 antibody (IP/WB, bands boxed). Lower levels of full length PTPRG is present in EtOH treated cells. The same antibody was used in WB analysis on the corresponding serum-free conditioned media precipitated with TCA/acetone (right panel, boxed). The ∼120 kDa PTPRG isoform was detectable in SFCM at higher levels in comparison with untreated cells. The first two lanes represent total cell lysate before IP and demonstrate equal amount of protein and comparable surface biotin labeling of the two samples.

WB of HepG2 cells (used routinely for a variety of biochemical and cell biological assays addressing hepatocyte functions [24]) confirmed the presence of both ∼120 and ∼90 kDa bands found in the human plasma and mouse liver. The full-length protein was undetectable in SFCM (**Fig. 5A**). The ∼120 kDa band and was strongly down-regulated by treatment of the cell with siRNA specific for PTPRG (**Fig. 5B**) further demonstrating the identity of the band and the specificity of the antibody used for its detection. We next investigated whether the ∼120 kDa isoform could represent a cleavage product derived from the full-length PTPRG protein. HepG2 cells were treated in serum free medium with 50μM of furin inhibitor or with GM6001 (Ilomastat), a matrix metalloprotease inhibitor. We found that only GM6001 inhibited the production of the cleaved forms (**Fig. 5C and D**).

### sPTPRG levels increase in the presence of liver injury

**Fig 6:**
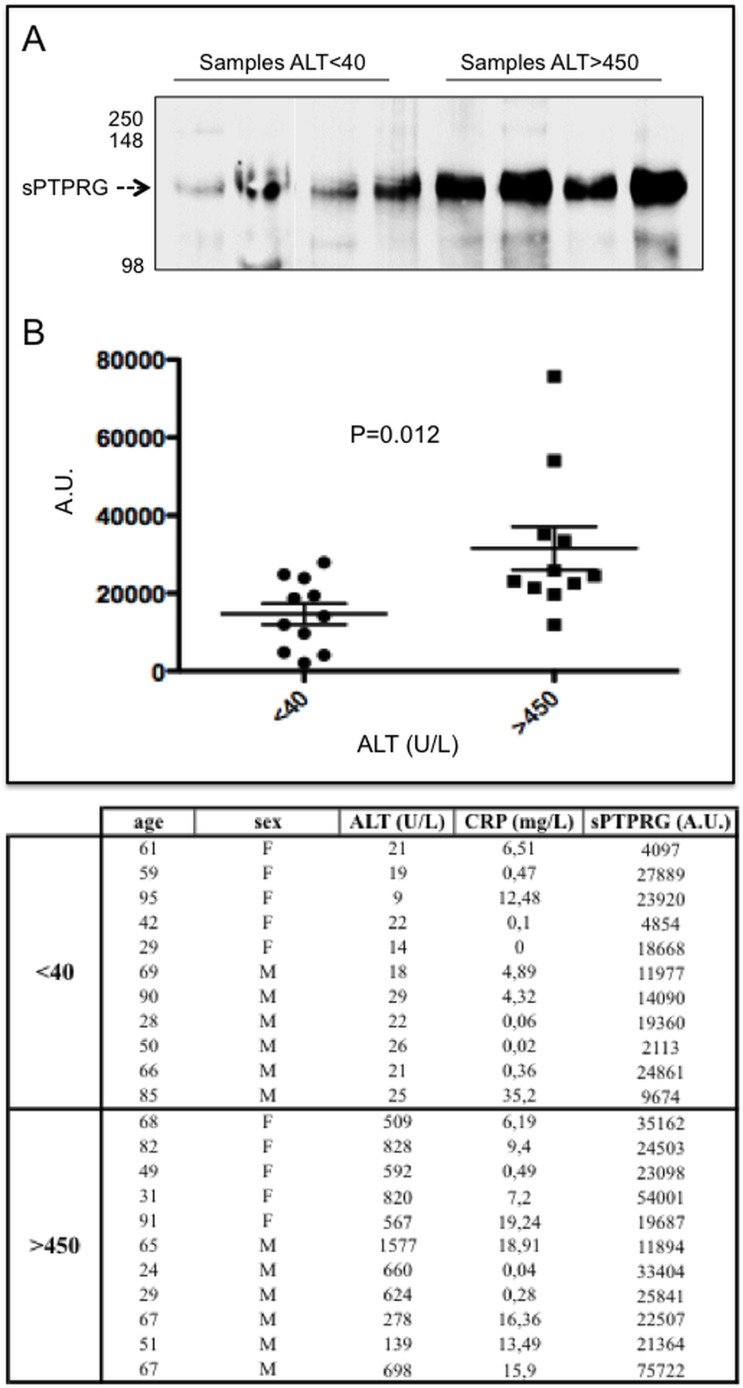
Relationships between liver damage and sPTPRG expression levels in human plasma. **Panel A**: representative immunoblotting of plasma (immunoprecipitation with CNBR/anti-P4 and WB with the same antibody) and samples from groups with ALT levels < 40 and > 450 U/L. Dashed arrow indicates the soluble PTPRG isoform of ∼120 kDa (sPTPRG). Panel B: Samples from individuals with Alanine aminotransferase (ALT) levels < 40 and > 450 U/L were evaluated for PTPRG expression measured as normalized values of arbitrary units of light emitted from immunoprecipitated serum samples separated by SDS-PAGE and immunoblotted with anti-P4 antibody (ECL detection system) as described in material and methods. Values of C-reactive protein (CRP, expressed in mg/L) are also shown. Unpaired Student t-test was performed on the data shown. The table summarizes the relevant clinical and biochemical data associated with the samples.

Given the identification of liver as a major source of sPTPRG we next evaluated whether its expression could be influenced by pathologic conditions affecting this organ. We first addressed the issue whether increase of sPTPRG could occur in the conditioned medium of cells incubated with ethanol, a well known cause of liver damage[25]. We exposed HepG2 cells for 16 hrs to sub-maximal dose (50 mM) of ethanol. We then performed cell surface and conditioned medium biotinylation followed by immunoprecipitation with anti-P4 antibody. The result shown in **Fig. 5E** show that the increase of sPTPRG in the conditioned medium after ethanol treatment is associated to a decrease in full length PTPRG present on the cell membrane immunoprecipitated from the same amount of cell lysate. This result suggests a link between liver damage and the presence of sPTPRG that we set to evaluate in clinical samples. Alanine aminotransferase (ALT), an intracellular enzyme normally present in many tissues, is considered a reference plasma biomarker of liver injury[26]. Plasma samples from two groups of individuals, one with ALT levels < 40U/mL (n = 11, age 61±23) and another with levels > 450 U/mL (n = 11, age 57±22) were selected for immunoprecipitation studies as an ELISA assay suitable for the extracellular domain is not yet available for sPTPRG. To normalize the results of densitometric analysis after WB, a reference control plasma sample was always processed in all the immunoprecipitation experiments. sPTPRG expression was significantly increased in plasma samples with the higher (>450U/mL) ALT levels (**Fig. 6**).

## Discussion

Blood plasma contains a number of potential biomarkers reflecting organ and tissue pathophysiology. Not only “classic” plasma proteins, but also cellular “leakage” proteins can virtually originate from any cell or tissue type in the body and can be released in the plasma. Moreover, thanks to its accessibility and to the wide number of different detectable molecular components reflecting the metabolism of cells in tissues, plasma may provide direct information on disease progression, on recovery from disease and on drug response. As such, the discovery of novel candidate protein biomarkers in plasma could open new avenue of investigation in this challenging field. In general, soluble and membrane-bound isoforms of the same protein that circulate in the blood plasma can be generated by: 1) separate biosynthetic pathways, either by alternative pre-mRNA splicing of a common transcript or by transcription of closely related but distinct genes; 2) posttranslational release of the extracellular domain of membrane proteins by hydrolytic cleavage of the membrane anchor. The latter appears to represent a convenient and common mechanism utilized by cells allowing multiplying the functional roles of proteins with a minimum investment in energy and structural complexity. Apparently, membrane proteins are specifically released by proteolysis in a directed and sometimes regulated manner to produce active, soluble protein forms. These include proteins that have sufficiently different structure and function to suggest that spatially limited proteolysis may constitute a general mechanism to release membrane proteins and generate soluble isoforms with defined functions[28,29]. In this study we have identified different PTPRG soluble isoforms present in human and mouse plasma including the full length PTPRG protein linked to the plasma membrane associated to cell-derived exosomes. The detection of a major fragment of ∼120 kDa by specific antibodies reacting with PTPRG extracellular domain suggest that this isoform may represent the complete ECD described by others[19]. Additional isoforms we detected in the human plasma and mouse serum include a ∼90 kDa fragment (also present in human/mouse plasma, murine pancreas, lung, liver and in both the cell lysates and SFCM of HepG2 cell line), and a ∼70 kDa protein, detected in all the perfused tissues analyzed except kidney. Considering the reactivity of mono/polyclonal antibodies we utilized in WB experiments we can conclude that the ∼90 kDa protein may be the ECD lacking a N-terminal fragment and possessing a short tail of the intracellular domain, as it reacts with an antibody directed against an epitope which is located in this region. A possibility exists that ∼120 kDa protein and the ∼90 co-immunoprecipitate under the experimental conditions used in our assays. This last interpretation is suggested by the observation that the association of E and P subunits of PTPRF (LAR), not disulfide bonded, was stable under standard cell lysis condition and was sufficiently strong to resist disruption during extended washing of the samples [10]. Also PTPRS was expressed in two subunits that were derived from a precursor protein by proteolytic processing and also in this case E and P subunits co-immunoprecipitate [30]. The identification of ∼120 and ∼90 kDa isoforms in HepG2 cells and in the liver of perfused mouse can support the hypothesis that sPTPRG could be enzymatically cleaved (both intracellularly as well as extracellularly) and then released in the plasma. This hypotheses is supported by the significantly lower expression of sPTPRG found in the plasma of individuals with ALT levels <40U/mL as compared to the higher sPTPRG present in the plasma of patients with ALT >450 U/mL. The results of cleavage experiments may indicate that although a furin recognition motif R*XX*R (aa 703–706) is present in the extracellular domain of PTPRG [31] its cleavage is not inhibited by a furin inhibitor but rather by a metalloprotease inhibitor suggesting a role for this class of enzymes in the processing of PTPRG.

Finally, the presence in murine tissues of a ∼70 kDa isoform, not detectable in plasma/serum, may suggest the presence also of an intracellular, immature form of a soluble protein, or of a fragment of the full length or ∼120 kDa isoform lacking part of the C-terminal portion of the ECD.

## Conclusion

Although the function of sPTPRG is currently unknown the identification of new, readily detectable plasmatic isoforms of PTPRG, whose levels increase in the presence of hepatocyte damage, represent a novel finding, prompt the investment in the development of dedicated assays and open a new avenue of research on the physiopathological role of this protein in the context of liver injury and/or in the regulation of PTPs-dependent signaling events.
